# Variants in *ZNRD1* Gene Predict HIV-1/AIDS Disease Progression in a Han Chinese Population in Taiwan

**DOI:** 10.1371/journal.pone.0067572

**Published:** 2013-07-09

**Authors:** Ying-Ju Lin, Yu-Ching Lan, Chien-Hui Hung, Ting-Hsu Lin, Shao-Mei Huang, Chiu-Chu Liao, Cheng-Wen Lin, Chih-Ho Lai, Ni Tien, Xiang Liu, Mao-Wang Ho, Wen-Kuei Chien, Jin-Hua Chen, Jen-Hsien Wang, Fuu-Jen Tsai

**Affiliations:** 1 Department of Medical Research, China Medical University Hospital, Taichung, Taiwan; 2 School of Chinese Medicine, China Medical University, Taichung, Taiwan; 3 Department of Health Risk Management, China Medical University, Taichung, Taiwan; 4 Graduate Institute of Clinical Medical Science, Chang-Gung University, Chiayi, Taiwan; 5 Department of Medical Laboratory Science and Biotechnology, China Medical University, Taichung, Taiwan; 6 Department of Microbiology, School of Medicine, China Medical University, Taichung, Taiwan; 7 Molecular Virology Section, Laboratory of Molecular Microbiology, National Institute of Allergy and Infectious Diseases, National Institutes of Health, Bethesda, Maryland, United States of America; 8 Section of Infectious Diseases, Department of Internal Medicine, China Medical University Hospital, Taichung, Taiwan; 9 Biostatistics Center, China Medical University, Taichung, Taiwan; 10 Department of Biotechnology, Asia University, Taichung, Taiwan; University of Amsterdam, Netherlands

## Abstract

Patients demonstrate notable variations in disease progression following human immunodeficiency virus (HIV) infection. We aimed to identify *ZNRD1* and *RNF39* genetic variants linked to AIDS progression. We conducted a genetic association study in HIV-1-infected Han Chinese patients residing in Taiwan. The clinical characteristics of 143 HIV-1-infected patients were measured, and patients were split into 2 groups: AIDS progression and AIDS non-progression. Genotyping of *ZNRD1* and *RNF39* was performed in all participants. We found that patients in the AIDS progression group had higher HIV-1 viral loads and lower CD4 cell counts than did patients in the AIDS non-progression group. The frequency of the AA genotype of *ZNRD1* (rs16896970) was lower in the AIDS progression group than in the AIDS non-progression group. Patients with AA genotypes had lower levels of HIV-1 viral loads and higher levels of CD4 cell counts than did patients with AG+GG genotypes. AIDS progression in patients with the AA group is significantly different from that in patients with the AG and GG groups by using Kaplan-Meier survival analysis. The hazard ratio for progression was lower in the AA group than in the AG and GG groups. We identified a SNP that contributes to AIDS progression in HIV-1-infected patients in this population. This SNP had a significant protective influence on AIDS progression, and polymorphisms of the *ZNRD1* gene may play a role in the pathogenesis of HIV-1 infection.

## Introduction

HIV/AIDS remains one of the most significant and challenging infectious diseases worldwide despite the introduction of antiretroviral therapy [1]. According to the UNAIDS report, there were approximately 34.0 million people living with HIV at the end of 2011. Approximately 2.5 million newly infected people were identified and 1.7 million people died from AIDS-related causes in 2011.

Patients show variable clinical outcomes in response to HIV infection. AIDS usually develops within 10 years of infection in those who do not receive any therapy. Among these patients, however, diverse disease progression rates are observed [2], [3], [4]. Some patients show rapid, regular, or slow disease progression, whereas others are completely asymptomatic for more than 15 years. This diversity of clinical outcomes is believed to result from complex interactions among the virus, host, and environment factors.

Genome-wide and RNA interference studies have been conducted to identify host cellular genes that affect HIV replication as well as disease progression [5], [6], [7], [8], [9], [10], [11], [12], [13], [14]. Among these identified candidate genes, a locus on human chromosome 6, close to the *ZNRD1* (zinc ribbon domain-containing 1) and *RNF39* (ring finger protein 39) genes, shows an interesting correlation with AIDS progression [11], [15]. Results from genome-wide siRNA screens also suggested that the ZNRD1 protein is required for HIV replication [8].

*ZNRD1*, which was cloned from the human MHC class I region [16], is found in a cluster with *RNF39*. The N-terminal domain of *ZNRD1* functions as an RNA polymerase, while the C-terminal domain (containing the zinc ribbon domain) functions as a transcription-associated protein. Recently, the ZNRD1 protein has been associated with the regulation of cell growth in gastric cancer and leukemia [17], [18]. *RNF39* is a poorly characterized gene, and the biological functions of the protein are not well known beyond its role in the early phase of synaptic plasticity in rats [19]. Furthermore, the role of *RNF39* in HIV-1 infection is poorly understood.

We conducted a genetic association study to identify *ZNRD1* and *RNF39* genetic variants associated with AIDS progression in HIV-1-infected Han Chinese patients residing in Taiwan.

## Patients and Methods

### Study Participants

The HIV-1 infection study is an observational study that was established in Middle Taiwan in 2001. Voluntary participants provided written informed consent and agreed to provide long-term follow-up clinical, immunovirological, and epidemiological data, and donated cryopreserved plasma and peripheral blood mononuclear cells. HIV-1-antibody-positive individuals were recruited, and HIV status was confirmed by quantitative HIV-1 RNA measurement (Roche COBAS TaqMan HIV-1 assay v2.0). CD4 and CD8 cell numbers were measured using a Coulter Epics® Profile II™ flow cytometer (Coulter Manual CD4 Count Kit; Coulter Manual CD8 Count Kit). HIV-1-positive individuals were followed until recently (June 2012). These participants were then divided into 2 further groups: CD4 count <350 cells/µl or <14% of total lymphocytes (AIDS progression group); and CD4 count ≧ 350 cells/µl or ≧ 14% of total lymphocytes (AIDS non-progression group). For AIDS progression analysis, the final sample of 143 HIV-1 patients were (a) naïve for antiretroviral treatment, (b) belonging to the Han Chinese ethnic group, and (c) willing to give blood samples for genotyping. The study was terminated in June 2012, resulting in an observation period (follow-up time) of approximately 2300 days (Table 1). The study was approved by the Institutional Review Board of China Medical University Hospital. All participants read and signed informed consent documents.

**Table 1:** Baseline characteristics of HIV-1 patients with or without AIDS progression in a Han Chinese population in Taiwan.

Variable	Patients with AIDS progression	Patients with AIDS-non progression	
**No. of participants**	90	53	
**Male sex, %**	91.1	90.6	
**Mean age at HIV-1 antibody positive (range), years** a	35.1 (21.3–56.1)	36.0 (23.9–54.7)	
**Total observation time since the person was** **examined HIV-1 sero-positive (range), days** b	2209.3 (1598–2961)	2242.1 (1652–3109)	
**Mean plasma HIV-1 viral load (range), log_10_ copies/mL** c	4.1 (2.6–5.8)	3.6 (2.6–4.8)	
**Mean CD4 count (range), cells/µL** d	379.0 (193–731)	643.9 (411–1213)	
**Mean CD8 count (range), cells/µL** e	1174.0 (379–2786)	1332.50 (488–3717)	

Statistical significance at *p*<0.05.

a The age with HIV-1 antibody positive means the age of the person when he/she was examined with the earlist positive for HIV-1 antibody result. The HIV-1 antibody positive results were from the database of the department of medical and laboratory examination of our hospital.

b *p*-value = 0.993 by using the unpaired Student t test. The total observation time is the duration between the lastest visiting date and the date when the person was examined with the earlist HIV-1 antibody positive result.

c *p*-value <0.0001 by using the unpaired Student t test. The HIV-1 viral load was measured in peripheral blood sampled in the first 3–27 months when he/she was examined with the earlist HIV-1 antibody positive result. Any measurements taken after the start of anti-retroviral therapy were not used in any analysis.

d *p*-value <0.0001 by using the unpaired Student t test. The CD4 count at enrollment was measured in peripheral blood sampled in the first 3–27 months when he/she was examined with the earlist HIV-1 antibody positive result. Any measurements taken after the start of anti-retroviral therapy were not used in any analysis.

e *p*-value = 0.351 by using the unpaired Student t test. The CD8 count at enrollment was measured in peripheral blood sampled in the first 3–27 months when he/she was examined with the earlist HIV-1 antibody positive result. Any measurements taken after the start of anti-retroviral therapy were not used in any analysis.

### Blood Sample Collection, Measurement of Cell Counts, and Plasma HIV-1 Viral Load

Blood was collected by venipuncture and peripheral blood mononuclear cells (PBMCs) were isolated by Ficoll-Histopaque (Sigma, 1077) density-gradient centrifugation and frozen until use. Plasma viral loads were measured by quantitative HIV-1 RNA nucleic acid-based sequence amplification (Roche COBAS TaqMan HIV-1 assay v2.0), with multiplex real-time PCR detection of sequences in the long terminal repeat and gag regions in the HIV-1 genome [20]. The number of viral RNA copies in plasma was below the arbitrarily chosen quantification test threshold of 40 copies/ml plasma. Plasma CD4 and CD8 cells were measured using a *Coulter Epics*® *Profile II*™ *flow cytometer*. Cell counting was performed according to the manufacturer’s guidelines (*Coulter* Manual *CD4 Count* Kit). Any measurements taken after the start of antiretroviral therapy were not used in any analysis.

### Detection of *ZNRD1* and *RNF39* Genetic Polymorphisms

**Figure 1:**
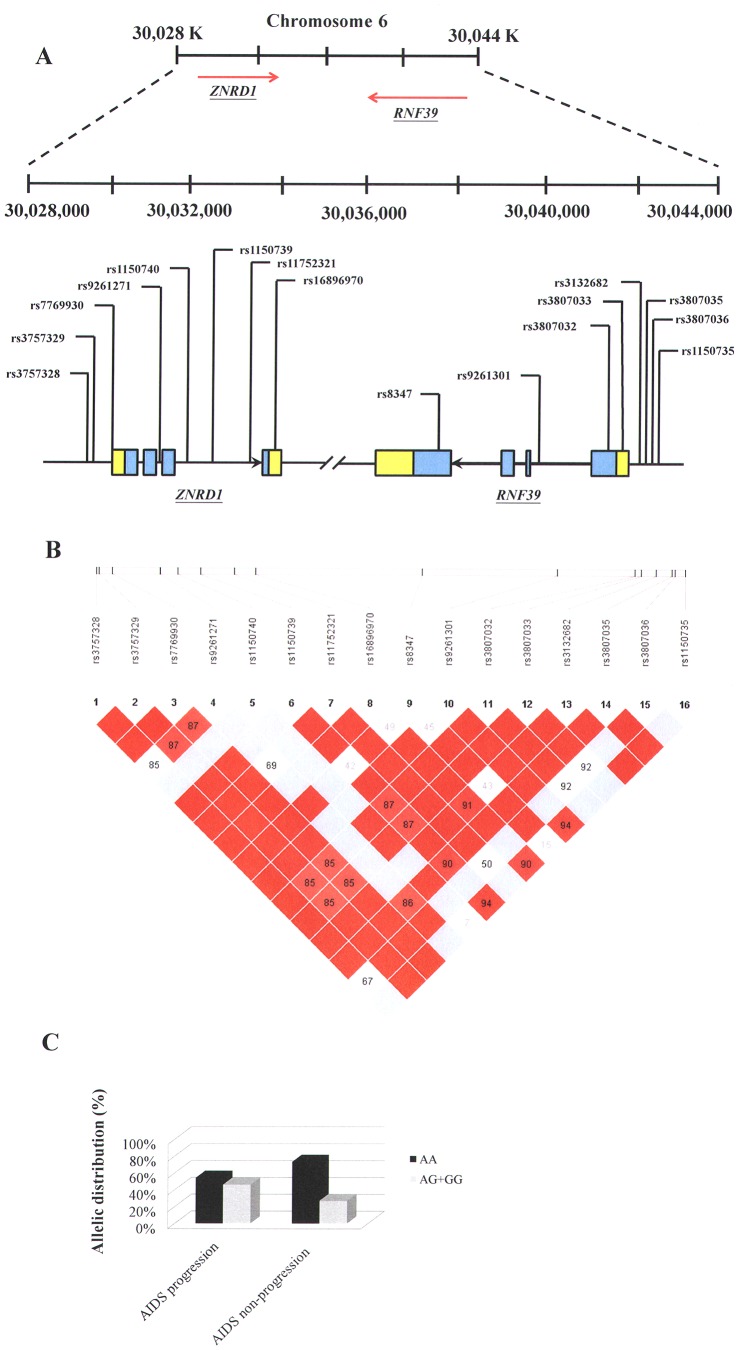
Single nucleotide polymorphisms (SNPs) analyzed and the linkage disequilibrium (LD) pattern of the *ZNRD1* and *RNF39* genes used in this study. **A:** Genomic location of SNPs present on chromosome 6p21. **B:** Linkage disequilibrium (LD) blocks in the *ZNRD1* and *RNF39* genes, estimated by using HAPLOVIEW software. Pairwise D’ values (%) are indicated in squares; red indicates linkage disequilibrium (D’ = 1, logarithm of odds (LOD) ≥2); blue indicate evidence of recombination (D’ = 1, LOD<2). **C:** Allelic distribution (%) of the significant SNP (rs16896970) in the AIDS progression and non-progression groups.

Eight single nucleotide polymorphisms (SNPs) from *ZNRD1* and 8 SNPs from *RNF39* were selected from the NCBI SNP database and HAPMAP website (Figure 1 A and Table 2) [21], [22], [23]. Selection criteria for including SNPs in the analysis were a minimum allele frequency of >0.05 in the Han Chinese population and a Hardy-Weinberg equilibrium (*p*>0.05). A summary of information on the SNPs in the *ZNRD1* and *RNF39* genes (location, position, rs number, and genotype) is presented in Table 2. Briefly, genomic DNA was extracted from peripheral blood leukocytes according to standard protocols (Genomic DNA kit; Qiagen). SNPs were genotyped using a custom-designed VeraCode GoldenGate Genotyping Assay System (Illumina) [24]; genotyping was performed as outlined at http://www.illumina.com/.

**Table 2:** Effects of *ZNRD1*and *RNF39* gene SNPs on AIDS progression of HIV-1 patients in a Han Chinese population in Taiwan.

Gene	Location	Position	SNP		Genotype	Patients with AIDS progression	Patients with AIDS-non progression	*p*-value	HR	95% CI
						Genotype frequency (no (%))	Genotype frequency (no (%))			
*ZNRD1*	5′ near gene	30,028,352	SNP1	rs3757328	GG	59 (67.8)	43 (82.7)	0.325	0.797	0.508–1.251
					AG+AA	28 (32.2)	9 (17.3)			
	5′ near gene	30,028,424	SNP2	rs3757329	AA	56 (64.4)	38 (73.1)	0.937	0.982	0.633–1.526
					AC+CC	31 (35.6)	14 (26.9)			
	5′ near gene	30,028,807	SNP3	rs7769930	AA	56 (64.4)	38 (73.1)	0.937	0.982	0.633–1.526
					AC+CC	31 (35.6)	14 (26.9)			
	intron	30,030,189	SNP4	rs9261271	TT	82 (94.3)	46 (88.5)	0.161	1.909	0.772–4.720
					AT+AA	5 (5.7)	6 (11.5)			
	intron	30,030,689	SNP5	rs1150740	CC	85 (97.7)	49 (94.2)	0.328	2.014	0.495–8.195
					AC+AA	2 (2.3)	3 (5.8)			
	intron	30,031,345	SNP6	rs1150739	AA	29 (34.1)	19 (36.5)	0.398	1.215	0.773–1.910
					AG+GG	56 (65.9)	33 (63.5)			
	intron	30,032,319	SNP7	rs11752321	CC	61 (70.1)	44 (84.6)	0.361	0.807	0.509–1.279
					CG+GG	26 (29.9)	8 (15.4)			
	3′ near gene	30,032,917	SNP8	rs16896970	AA	47 (54.0)	38 (73.1)	***0.006***	***0.551***	***0.359–0.845***
					AG+GG	40 (46.0)	14 (26.9)			
*RNF39*	3′ near gene	30,037,686	SNP1	rs8347	GG	33 (40.7)	17 (35.4)	0.682	0.911	0.584–1.422
					AG+AA	48 (59.3)	31 (64.6)			
	intron	30,041,559	SNP2	rs9261301	GG	29 (33.3)	18 (34.6)	0.331	1.249	0.797–1.957
					AG+AA	58 (66.7)	34 (65.4)			
	5′ near gene	30,043,779	SNP3	rs3807032	GG	57 (65.5)	40 (76.9)	0.617	0.893	0.574–1.391
					CG+CC	30 (34.5)	12 (23.1)			
	5′ near gene	30,043,955	SNP4	rs3807033	GG	57 (65.5)	40 (76.9)	0.617	0.893	0.574–1.391
					AG+AA	30 (34.5)	12 (23.1)			
	5′ near gene	30,044,388	SNP5	rs3132682	CC	28 (32.2)	17 (32.7)	0.299	1.271	0.808–1.999
					CG+GG	59 (67.8)	35 (67.3)			
	5′ near gene	30,044,827	SNP6	rs3807035	GG	54 (62.1)	35 (67.3)	0.729	1.080	0.699–1.668
					AG+AA	33 (37.9)	17 (32.7)			
	5′ near gene	30,044,914	SNP7	rs3807036	GG	83 (95.4)	46 (88.5)	0.131	2.169	0.794–5.930
					AG+AA	4 (4.6)	6 (11.5)			
	5′ near gene	30,045,199	SNP8	rs1150735	GG	47 (54.0)	28 (53.8)	0.881	1.033	0.676–1.578
					AG+AA	40 (46.0)	24 (46.2)			

HR, hazard ratio; CI, confidental interval.

The *p* values were adjusted by using a multiple testing correction method for genetic association studies using correlated SNP (*Genet Epidemiol.* 2008 May;32(4):361–369.).

Bold, emphasizing statistical significance was considered as *p* value <0.0083 (0.05/6). *p*-values were obtained by using Cox model analysis.

Primers and probes were designed and created using Custom VeraCode GoldenGate Genotyping Assay System software. Multiplex PCRs were performed with 144-plex VeraCode SNP arrays for 480 samples, and genotype analyses were performed using custom 96-plex SAM arrays for 96 samples. Genotype calls were automatically generated using GenCall software version 3.1.3. We assessed the 8 VeraCode runs individually for intra-plate inconsistencies (e.g., variation in fluorescent intensities). Genotype cluster plots generated by individual VeraCode and SAM assays were visually inspected for call quality. Plots that appeared to be “unusually” clustered (i.e., unlike the predicted spread in terms of software-generated HWE or distance between clusters [θ]) were investigated further by selecting samples via direct Sanger sequencing for genotype confirmation. Samples were sequenced using Big Dye Terminator v3.1 (AB, Foster City, CA, USA) according to the manufacturer’s guidelines, and sequenced with an AB 3730 genetic analyzer.

### Statistical Analyses

**Figure 2:**
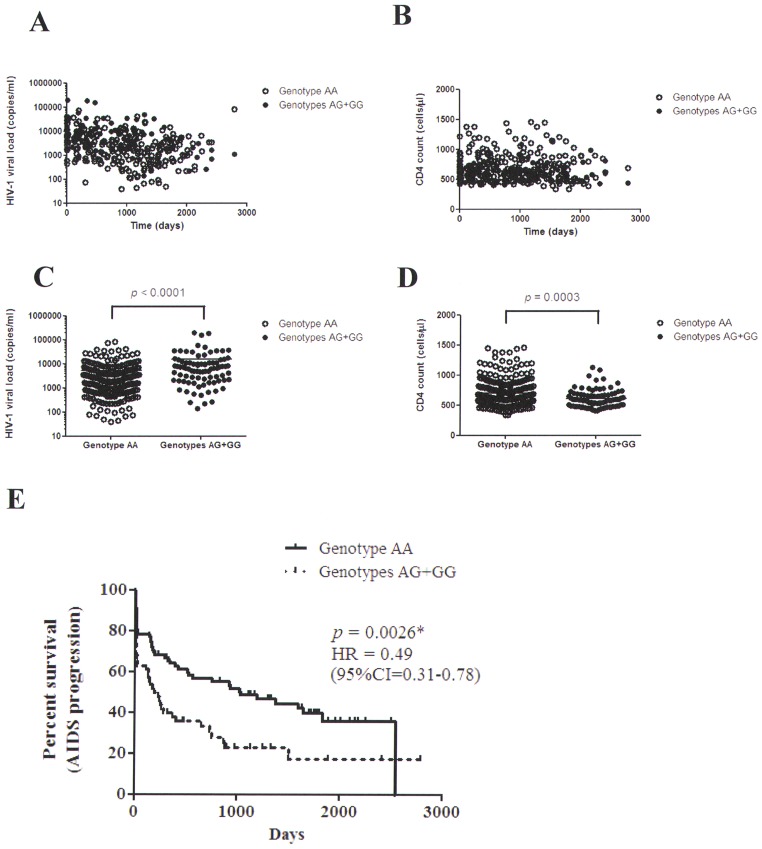
Comparison of HIV-1 viral loads, CD4 counts and percent survivial analysis between the significant SNP (rs16896970) genotypes. **A:** Measurements of HIV-1 viral load after infection were shown between the AA and AG+GG genotypes. **B:** Measurements of CD4 count after infection were shown between the AA and AG+GG genotypes. **C:** Analysis between HIV-1 viral load and the AA and AG+GG genotypes (*p*<0.0001; the unpaired Student t test). **D:** Analysis between CD4 count and the AA and AG+GG genotypes (p<0.0001; the unpaired Student t test). **E:** Kaplan-Meier survival analysis was performed to assess the difference between the AA and AG+GG genotypes (*p* = 0.0026, log-rank test).

Genotypes were obtained by direct count, followed by allele frequency calculations (Table 2). Chi-square tests were used to determine differences in categorical variables, and odds ratios (OR) and 95% confidence intervals (CI) were calculated for the factors under consideration. In addition to chi-square tests, *p* values were calculated using the Minitab program; the *p* values were adjusted by using a multiple testing correction method for genetic association studies using correlated SNP [25]. Statistical significance was considered as *p* value <0.0083 (0.05/6). The unpaired Student t test was used for comparison between groups (Figure 2 C and D). For analysis of haplotype blocks (Figure 1B), we used the Lewontin D′ measure to estimate the intermarker coefficient of linkage disequilibrium (LD) of our HIV-1 patients by using the HAPLOVIEW software [23]. The confidence interval of LD was estimated using a resampling procedure and was used to construct the haplotype blocks [26]. A Kaplan-Meier survival analysis (log-rank test) was performed to assess the difference in CD4 decline (a decline of CD4 counts to <350 cells/µl) among the *ZNRD1* rs16896970 genotype groups (Figure 2 E), and Cox regression was used to acquire hazard ratios. All statistical analyses were performed using SPSS (v12.0) for Windows, and graphs were generated using GraphPad Prism version 5.01 for Windows (GraphPad Software, San Diego, California, USA).

## Results and Discussion

To identify genetic variants linked to AIDS progression, we genotyped the *ZNRD1* and *RNF39* genes in 143 HIV-1-infected patients (90 individuals with AIDS progression and 53 individuals without AIDS progression) (Tables 1 and 2). The characteristics and clinical profiles of patients in the AIDS progression and AIDS non-progression groups are summarized in Table 1. Statistically significant differences in mean plasma HIV-1 viral loads and CD4 cell counts were found between the 2 groups (*p*<0.0001) (Table 1). Patients in the AIDS progression group had higher HIV-1 viral loads and lower CD4 cell counts than did the patients in the non-progression group.

The genetic location of *ZNRD1* and *RNF39* is shown in Figure 1; all SNPs were in Hardy-Weinberg equilibrium and had a successful genotyping frequency of >99%. The linkage disequilibrium structure of the *ZNRD1* and *RNF39* genomic regions was established, with 2 haplotype blocks determined (Figure 1 B). The first block contained 4 SNPs and included 3 *ZNRD1* exons. The second block contained 10 SNPs and included the last exon of the *ZNRD1* gene and 4 *RNF39* exons (Figure 1 B). Cox regression survival analysis showed that there were associations between genetic variants of *ZNRD1* and *RNF39* and AIDS progression (Table 2). As shown, statistically significant differences were observed for the *ZNRD1* (rs16896970) genetic variant (*p = *0.006; a relative hazard of 0.551 (95% CI: 0.359–0.845); Table 2). The AA major genotype of *ZNRD1* (rs16896970) was 54% for the AIDS progression group and 73.1% for the AIDS non-progression group, respectively (*p* = 0.026) (Figure 1 C). These results suggest that this SNP had a significant protective influence on progression to AIDS. Patients with the AA genotype tend to develop slower progression to AIDS than the patients who don’t have this genotype.

We also compared HIV-1 viral loads and CD4 cell counts between genotypes (Figure 2). HIV-1 viral load and CD4 cell count measurements were recorded during the observation period (Figure 2 A and B). There was an overall significant difference in viral loads and CD4 cell counts between genotypes (*p*<0.0001 for HIV-1 viral loads; *p = *0.0003 for CD4 cell counts) (Figure 2 C and D). Patients with the AA genotype had lower HIV-1 viral loads and higher CD4 cell counts than did the patients with the AG+GG genotype. AIDS progression in patients with the AA group is significantly different from that in patients with the AG and GG groups by using Kaplan-Meier survival analysis (*p = *0.0026; Figure 2 E). The result suggests that patients with the AA genotypes may associate with delayed AIDS progression within about 2600 days after infection. The hazard ratio for AIDS progression in the AA group compared with the AG and GG groups was 0.49 (95% confidence interval [CI] 0.31–0.78, *p = *0.0026).

In this study, we identified a SNP that contributes to AIDS progression in HIV-1-infected Han Chinese patients residing in Taiwan, in agreement with the results of genome-wide screens [8], [11], [27] in patients with the Caucasian genetic background. We observed that patients in the AIDS progression group had higher HIV-1 viral loads and lower CD4 cell counts than did patients in the AIDS non-progression group. Furthermore, we showed that there was a statistically significant difference between these 2 groups in the frequency of the *ZNRD1* (rs16896970) genetic variant. The AA genotype was more frequent in the AIDS non-progression group, and patients with this genotype had lower HIV-1 viral loads and higher CD4 counts than did those in the progression group. Our results suggest that this SNP has a significant protective influence, and polymorphisms of the *ZNRD1* gene may play a role in the pathogenesis of HIV-1 infection.

The protective SNP is located at the 3′UTR of the *ZNRD1* gene in the MHC region. Previous studies have investigated the correlation between genes of the MHC region and the outcome of HIV-1 infection [11], [13], [28], [29]. Of these, some studies have revealed inherent genetic complexity and high linkage disequilibrium of this region, disputing the effect of *ZNRD1* on AIDS progression [28], [30]. However, a genome-wide functional screen has suggested ZNRD1 protein is required for HIV-1 replication [8]. Ballana et al also reported that HIV-1 replication was impaired by ZNRD1 down-regulation by siRNA or shRNA at the transcription level in both lymphoid and non-lymphoid cells [31]. In agreement of these two studies, our supplementary data (Figure S2) also suggested that the inhibition of HIV-1 replication was also observed by *ZNRD1* RNA interference-mediated silencing in Jurkat cells using HIV-1 p24 ELISA detection. Furthermore, no inhibition of HTLV-1 replication was observed by *ZNRD1* RNA interference-mediated silencing in Jurkat cells using HTLV-1 p19 ELISA detection, suggesting that ZNRD1 protein may be required and specific for HIV-1 replication. In addition, *ZNRD1* was associated with AIDS progression even in individuals without *HLA-A10* [27]. Also, ZNRD1 has a strong statistically significant correlation with the Long-Term Non-Progressors (LTNP) phenotype (rs1048412; *p* = 0.0004), independently of *HLA-A10* [31]. We also download the SNPs data and the linkage disequilibrium (LD) structure of the Chinese (CHB) population from the HAPMAP website (Figure S3). There SNPs are rs3823339, rs3757328, rs3757329, rs7769930, rs9261271, rs1150740, rs1150739, rs11752321, rs16896970, rs8347, rs9261301, rs3807032, rs3807033, rs3132682, rs3807035, rs3807036 and rs1150735. Among these SNPs, a high LD between HLA-A*2601 (one of the serotypes of *HLA-A10*) and the SNP- rs3823339 has been reported in the CHB population [32]. As shown in Figure S3, no obvious high LD between rs3823339 and the *ZNRD1* and *RNF39* SNPs were observed. These results suggested that the HLA-A10 allele is not in high LD with the *ZNRD1* and *RNF39* SNPs. And there was no sufficient evidence to assign responsibility for the association.

Unlike previous studies, our study adopted a focused approach on individual polymorphisms around the *ZNRD1* and *RNF39* genes. The linkage disequilibrium structure of these 2 genes was determined, with 2 haplotype blocks determined. The 3′UTR of the *ZNRD1* gene is putatively involved in posttranscriptional mRNA regulation [33], [34]. In addition, the SNP we identified (rs16896970) showed a linkage disequilibrium with the SNP (rs3188482) (Table S1; D′ = 0.956), and the SNP (rs3188482) showed a linkage disequilibrium with the SNP (rs1048412) [31]. *ZNRD1* expression has previously been shown to be significantly associated with the SNP (rs3188482) (*p = *0.000006546) (http://app3.titan.uio.no/biotools/tool.php?app=snpexp). To investigate the correlation of the SNP rs16896970 genotypes with the *ZNRD1* expression, we measured *ZNRD1* mRNA levels by real-time quantitative PCR in peripheral blood mononuclear cells. As shown, the major allele homozygotes (AA genotypes) tend to express lower levels of *ZNRD1*, compared with the other individuals with AG+GG genotypes (*p* = 0.043; Figure S1).

*ZNRD1* encodes 1 subunit of an RNA polymerase I [16]. The blockade of RNA polymerase I is associated with the distribution of the HIV-1 regulatory protein Rev, causing dysregulation of transportation from the nucleolus to the cytoplasm [35], [36]. Rev is known to be localized in the nucleolus (where the RNA polymerase I transcribes ribosomal RNA). Rev participates in the export of unspliced/partially spliced viral RNAs from the nucleus [37]. ZNRD1 protein may interfere with the processing of HIV-1 viral RNA transcripts by the HIV-1 Rev protein, and may thus restrict HIV-1 replication.

In conclusion, we identified new genetic variants that are linked to AIDS progression in Taiwanese HIV-1 patients with a Han Chinese ethnic background, supporting the idea that host genetic factors may interfere with HIV-1 replication as well as AIDS progression. Our data also suggest the involvement of genetic variation in *ZNRD1* with AIDS progression. Research using larger cohorts is required to confirm these results. These findings emphasize the importance of studying individuals who have a range of genetic backgrounds as a guide to fighting HIV-1 infections.

## Supporting Information

Figure S1 **ZNRD1 mRNA expression levels in peripheral blood mononuclear cells between the** ***ZNRD1*** **SNP (rs16896970) genotypes.** The relative *ZNRD1* expression was detected by real-time RT-PCR, and expression from individuals with AG+GG genotypes was compared to that from individuals with AA genotypes. The relative expression levels were expressed as *ZNRD1* mRNA/HPRT mRNA ratio.
(TIF)

Figure S2 **Inhibition of HIV-1 replication but not HTLV-1 replication by** ***ZNRD1*** **RNA interference-mediated silencing in Jurkat cells.** **A:** Western blot of ZNRD1 in Jurkat cells transfected with siRNA targeting *ZNRD1* RNA transcript. **B:** HIV-1 p24 antigen ELISA detection of the culture supernatant in Jurkat cells transfected with individual siRNAs (Sramble or siZNRD1) and HIV-1 pNL4-3 clone. **C:** HTLV-1 p19 antigen ELISA detection of the culture supernatant in Jurkat cells transfected with individual siRNAs (Sramble or siZNRD1) and HTLV-1 K30 clone.
(TIF)

Figure S3 **Linkage disequilibrium (LD) structure of** HLA-A*2601 **tag SNP-rs3823339 (in CHB population;** ***Nat Genet.*** **2006; 38(10):1166–1172.),** ***ZNRD1*** **and** ***RNF39*** **gene SNPs.** Relative position of genes is based on NCBI Buil 36. Pairwise LD plots of the estimated statistics of the square of the correlation coefficient (r^2^) are illustrated with Haploview software. The values in each diamond, which indicate the LD relationship between each pair of SNPs, were derived from genotypes in the Han Chinese from HAPMAP website. Red diamonds without a number represent r^2^ = 1.
(TIF)

Table S1 **Analysis of LD among SNPs.**
(DOCX)
