# Supplementary figures and images for: Variants in ZNRD1 Gene Predict HIV-1/AIDS Disease Progression in a Han Chinese Population in Taiwan
Source: PLoS One. 2013 Jul 9;8(7):e67572. doi: 10.1371/journal.pone.0067572 (PMC3706582; doi:10.1371/journal.pone.0067572)

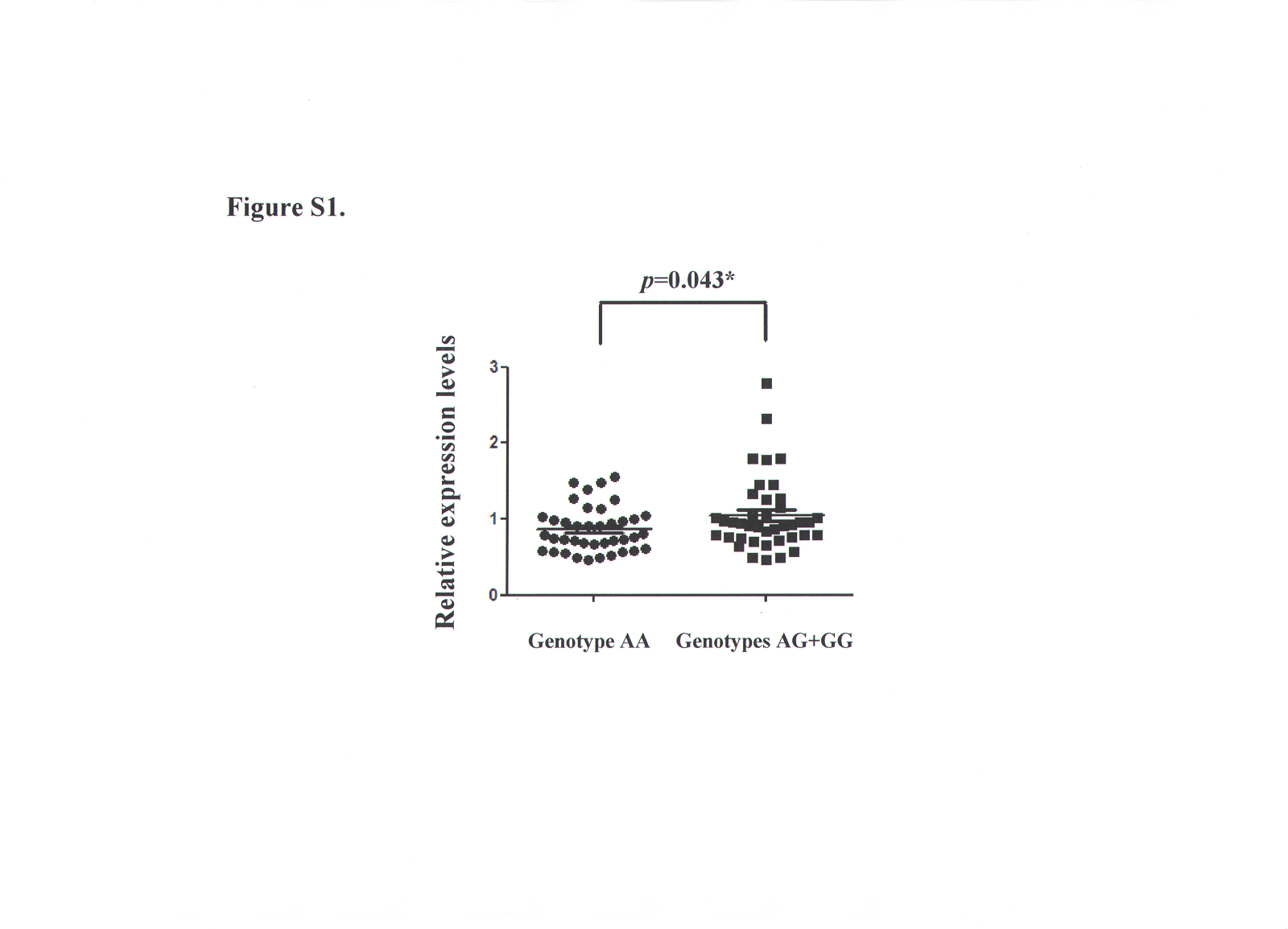

Supplement: Figure S1 — ZNRD1 mRNA expression levels in peripheral blood mononuclear cells between the ZNRD1 SNP (rs16896970) genotypes. The relative ZNRD1 expression was detected by real-time RT-PCR, and expression from individuals with AG+GG genotypes was compared to that from individuals with AA genotypes. The relative expression levels were expressed as ZNRD1 mRNA/HPRT mRNA ratio. (TIF) [file pone.0067572.s001.tif]

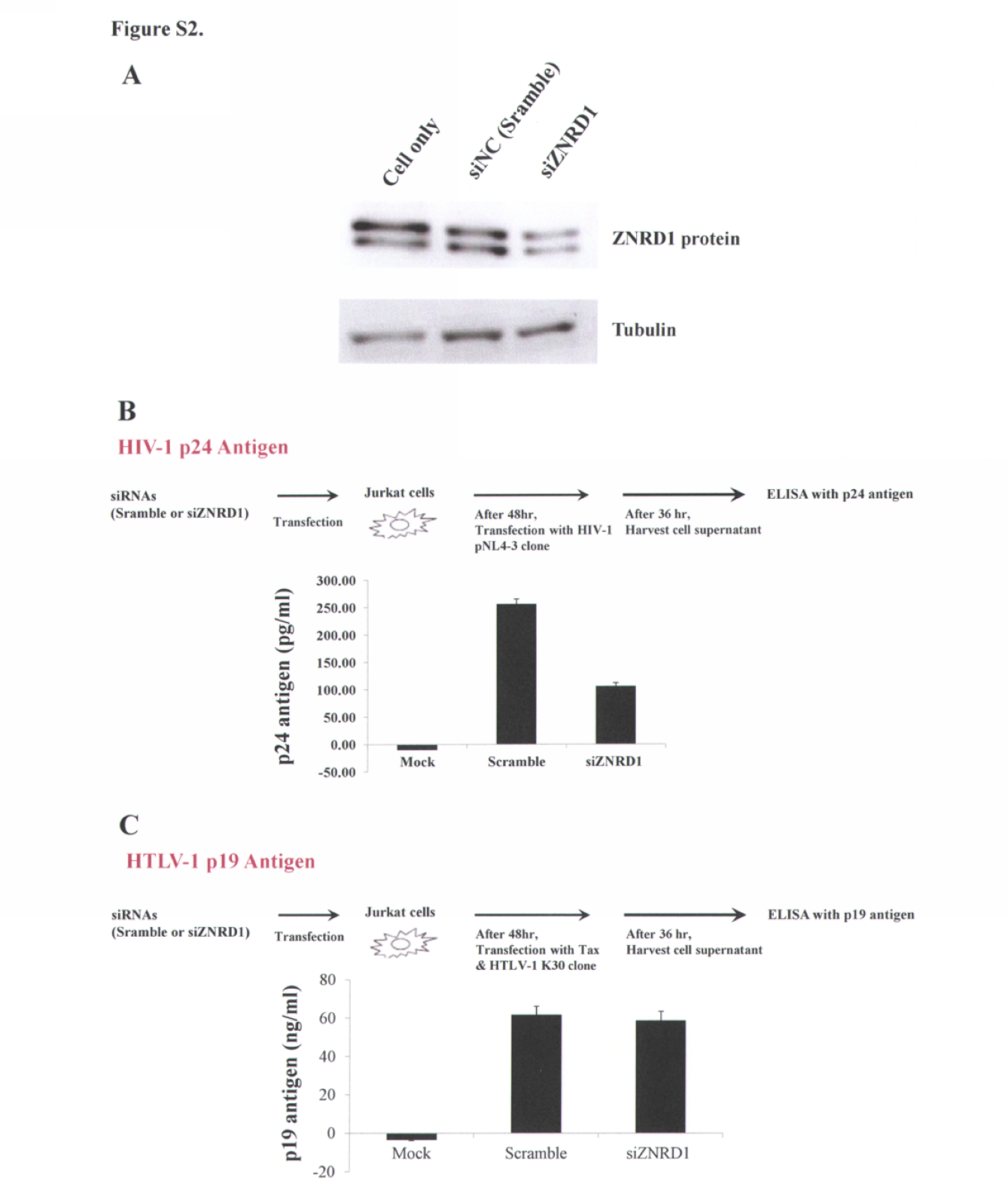

Supplement: Figure S2 — Inhibition of HIV-1 replication but not HTLV-1 replication by ZNRD1 RNA interference-mediated silencing in Jurkat cells. A: Western blot of ZNRD1 in Jurkat cells transfected with siRNA targeting ZNRD1 RNA transcript. B: HIV-1 p24 antigen ELISA detection of the culture supernatant in Jurkat cells transfected with individual siRNAs (Sramble or siZNRD1) and HIV-1 pNL4-3 clone. C: HTLV-1 p19 antigen ELISA detection of the culture supernatant in Jurkat cells transfected with individual siRNAs (Sramble or siZNRD1) and HTLV-1 K30 clone. (TIF) [file pone.0067572.s002.tif]

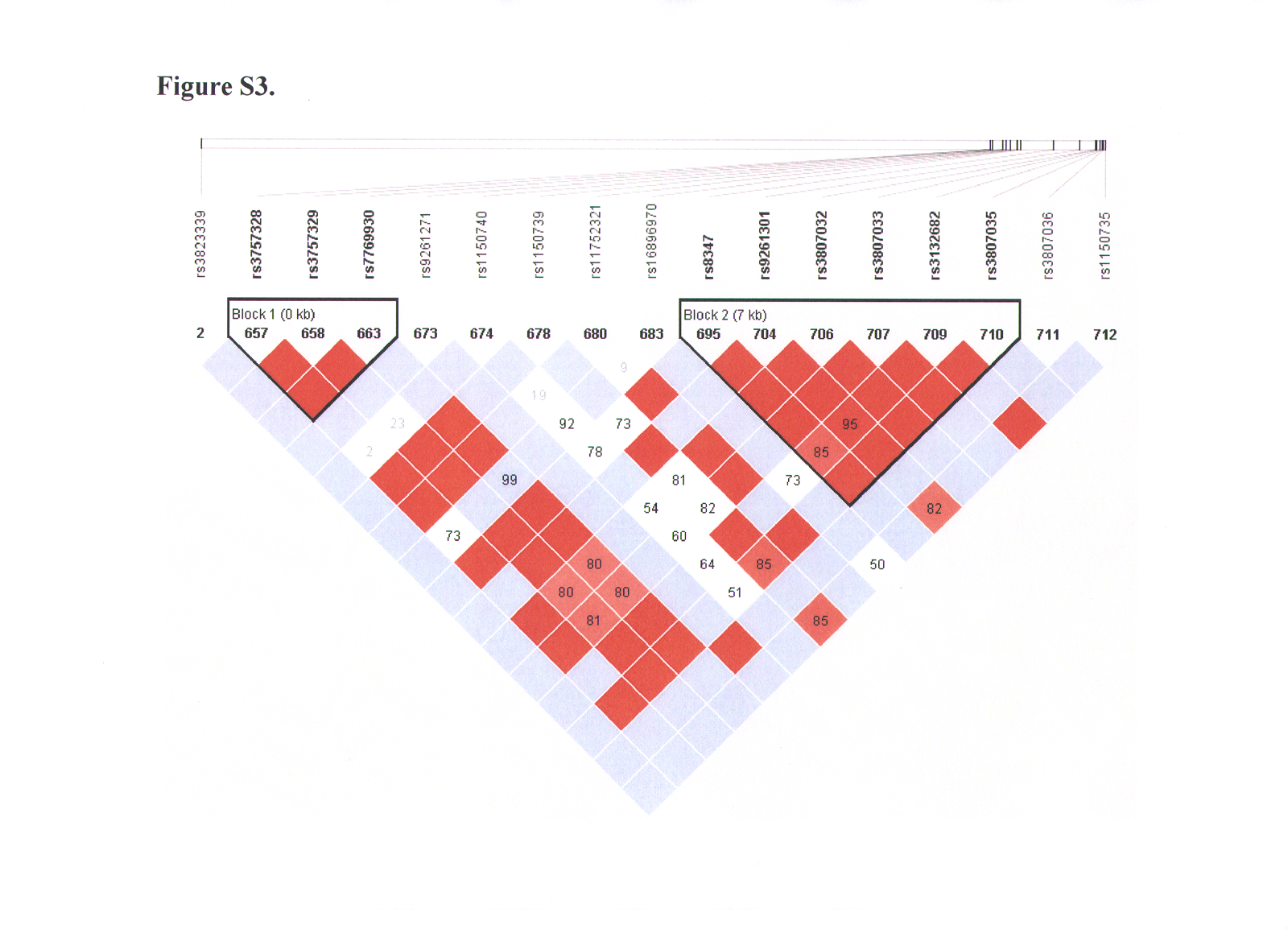

Supplement: Figure S3 — Linkage disequilibrium (LD) structure of HLA-A*2601 tag SNP-rs3823339 (in CHB population; Nat Genet. 2006; 38(10):1166–1172.), ZNRD1 and RNF39 gene SNPs. Relative position of genes is based on NCBI Buil 36. Pairwise LD plots of the estimated statistics of the square of the correlation coefficient (r2) are illustrated with Haploview software. The values in each diamond, which indicate the LD relationship between each pair of SNPs, were derived from genotypes in the Han Chinese from HAPMAP website. Red diamonds without a number represent r2 = 1. (TIF) [file pone.0067572.s003.tif]
