# Supplementary material for: Variants in ZNRD1 Gene Predict HIV-1/AIDS Disease Progression in a Han Chinese Population in Taiwan
Source: PLoS One. 2013 Jul 9;8(7):e67572. doi: 10.1371/journal.pone.0067572 (PMC3706582; doi:10.1371/journal.pone.0067572)
Supplement: Table S1 — Analysis of LD among SNPs. (DOCX) [file pone.0067572.s004.docx]

|  |  |  |  |  |
| --- | --- | --- | --- | --- |
|  |  |  |  |  |
|  |  |  |  |  |
|  | **Table S1. Analysis of LD among SNPs** | | | |
|  |  |  |  |  |
|  |  |  |  |  |
|  | ***Locus 1*** | ***Locus 2*** | ***HapMap Data^a^*** | |
|  |  |  |  |  |
|  |  |  | ***D'*** | ***LOD*** |
|  |  |  |  |  |
|  |  |  |  |  |
|  | rs3188482 | rs16896970 | 0.956 | 0.22 |
|  |  |  |  |  |
|  |  |  |  |  |
|  | D' = absolute value of Lewontin's D prime. | | | |
|  | ^a^The values of D' and LOD were obtained from the JPT plus CHB population of HAPMAP website. | | | |
|  |  |  |  |  |
|  |  |  |  |  |
